# Supplementary material for: T cell deficiency precipitates antibody evasion and emergence of neurovirulent polyomavirus
Source: eLife. 2022 Nov 7;11:e83030. doi: 10.7554/eLife.83030 (PMC9674346; doi:10.7554/eLife.83030)
Supplement: Supplementary file 1. — Identity, location, and frequency of detected VP1 mutations. Superscripted numbers indicate the VP1 loop in which the mutations are located (1: BC, 2: DE, 3: EF, 4: HI). Deletions are indicated by a “Δ” followed by the deleted residues; the identity of the deleted amino acids is indicated in “()”. The duplication of a residue is indicated with “dup.” The presence of two mutations in a virus is indicated with “+”. Sets of mutations separated by “and” indicate that both of the listed mutant viruses were isolated from the same mouse. [file elife-83030-supp1.docx]

| **Condition** | **VP1 Mutation** | **Loop** | **# of mice** |
| --- | --- | --- | --- |
| **IgG** | **E68K^1^ + Δ297(H)^4^**  **and**  **N149K^2^ + Δ297(H)^4^** | **BC^1^ + HI^4^**  **and**  **DE^2^ + HI^4^** | **1** |
|  | **E87K^1^ + Δ297(H)^4^** | **BC^1^ + HI^4^** | **1** |
|  | **E91V^1^ + Δ297(H)^4^** | **BC^1^ + HI^4^** | **1** |
|  | **Δ145-150(TDTVNT) + Δ294(Y)^4^** | **DE^2^ + HI^4^** | **1** |
|  | **E187K^3^ + Δ297(H)^4^** | **EF^3^ + HI^4^** | **1** |
|  | **Δ294(Y)^4^** | **HI^4^** | **1** |
|  | **Δ295(D)^4^** | **HI^4^** | **2** |
|  | **D295N^4^ + Δ296(F)^4^** | **HI^4^** | **1** |
|  | **V296F^4^** | **HI^4^** | **1** |
| **αCD4 + αCD8β** | **I79S^1^ + Δ295(D)^4^**  **and**  **N80K^1^ + Δ295(D)^4^** | **BC^1^ + HI^4^** | **1** |
|  | **K151dup^2^ + Δ297(H)^4^** | **DE^2^ + HI^4^** | **1** |
|  | **Δ292(R)^4^ + Y294H^4^** | **HI^4^** | **1** |
|  | **Y294D^4^** | **HI^4^** | **1** |
|  | **Δ295(D)^4^** | **HI^4^** | **7** |
|  | **D295A^4^ + Δ297(H)^4^** | **HI^4^** | **2** |
|  | **D295N^4^ + Δ297(H)^4^** | **HI^4^** | **1** |
| **αCD4** | **E68K^1^ + Δ297(H)^4^** | **BC^1^ + HI^4^** | **1** |
|  | **E87K^1^ + Δ297(H)^4^** | **BC^1^ + HI^4^** | **1** |
|  | **Δ147-148(TV)^2^ + Δ297(H)^4^**  **and**  **D295N^4^ + Δ297(H)^4^** | **DE^2^ + HI^4^**  **and**  **HI^4^** | **1** |
|  | **N293K^4^ + Δ297(H)^4^** | **HI^4^** | **2** |
|  | **N293K^4^ + Δ298(H)^4^** | **HI^4^** | **2** |
|  | **Δ295(D)^4^** | **HI^4^** | **2** |
|  | **D295A^4^ + Δ297(H)^4^** | **HI^4^** | **1** |
|  | **D295N^4^ + Δ297(H)^4^** | **HI^4^** | **1** |
| **αCD8β** | **Δ295(D)^4^** | **HI^4^** | **4** |
