# Supplementary material for: T cell deficiency precipitates antibody evasion and emergence of neurovirulent polyomavirus
Source: eLife. 2022 Nov 7;11:e83030. doi: 10.7554/eLife.83030 (PMC9674346; doi:10.7554/eLife.83030)
Supplement: Supplementary file 2. — Sequences of oligonucleotides used for site-directed mutagenesis, cloning, qPCR, and sequencing. [file elife-83030-supp2.docx]

| Designation | Reference | Sequence 5’-3’ |
| --- | --- | --- |
| Δ292/Y294H Forward | This paper | GGGAAGCCCTCTCCAGTGATGGACATCATGGTTTGTAACTCTCCAGCCCATTATATC |
| Δ292/Y294H Reverse | This paper | GATATAATGGGCTGGAGAGTTACAAACCATGATGTCCATCACTGGAGAGGGCTTCCC |
| Y294D Forward | This paper | GGGAAGCCCTCTCCAGTGATGGACATCATCGTTTCTTGTAACTCTCCAGCCC |
| Y294D Reverse | This paper | GGGCTGGAGAGTTACAAGAAACGATGATGTCCATCACTGGAGAGGGCTTCCC |
| D295A/Δ297 Forward | This paper | GGGAAGCCCTCTCCAGTGGACAGCATAGTTTCTTGTAACTCTCCAGCCCATTATATC |
| D295A/Δ297 Reverse | This paper | GATATAATGGGCTGGAGAGTTACAAGAAACTATGCTGTCCACTGGAGAGGGCTTCCC |
| D295N/Δ297 Forward | This paper | CTGGGAAGCCCTCTCCAGTGGACATTATAGTTTCTTGTAACTCTCCAGCCC |
| D295N/Δ297 Reverse | This paper | GGGCTGGAGAGTTACAAGAAACTATAATGTCCACTGGAGAGGGCTTCCCAG |
| D295N Forward | This paper | GGGAAGCCCTCTCCAGTGATGGACATTATAGTTTCTTGTAACTCTCCAGCCC |
| D295N Reverse | This paper | GGGCTGGAGAGTTACAAGAAACTATAATGTCCATCACTGGAGAGGGCTTCCC |
| Δ297 Forward | This paper | GGGAAGCCCTCTCCAGTGGACATCATAGTTTCTTGTAACTCTCCAGCCCATTATATC |
| Δ297 Reverse | This paper | GATATAATGGGCTGGAGAGTTACAAGAAACTATGATGTCCACTGGAGAGGGCTTCCC |
| VP1 TA cloning Forward | This paper | GGTCAACATAGCGCGTCATA |
| VP1 TA cloning Reverse | This paper | CCAGTTGAAATCTGGCATCC |
| VP1 sequencing | This paper | TACACTCTAACCTCCTCTACCTG |
| LT DNA qPCR Forward | Wilson et al., 2012 | CGCACATACTGCTGGAAGAAGA |
| LT DNA qPCR Reverse | Wilson et al., 2012 | TCTTGGTCGCTTTCTGGATACAG |
| LT DNA qPCR probe | Wilson et al., 2012 | ATCCTTGTGTTGCTGAGCCCGATG |
| LT mRNA qPCR Forward | Maru et al., 2017 | AGGAATTGAACAGTCTCTGGG |
| LT mRNA qPCR Reverse | Maru et al., 2017 | GTCATCGTGTAGTGGACTGTG |
| LT mRNA qPCR probe | Maru et al., 2017 | AACCGGCTTCCAGGGCTCT |
| VP1 Amplification Forward | Lauver et al., 2020 | CGACCCCTTGAAGGACATATGTGAA |
| VP1 Amplification Reverse | Lauver et al., 2020 | CACCTACTTGGGCAACAGTCA |
