# Supplementary material for: T cell deficiency precipitates antibody evasion and emergence of neurovirulent polyomavirus
Source: eLife. 2022 Nov 7;11:e83030. doi: 10.7554/eLife.83030 (PMC9674346; doi:10.7554/eLife.83030)
Supplement: Supplementary file 3. — Statistical tests used and exact p values for the comparisons of data presented in each figure as indicated. [file elife-83030-supp3.docx]

| **Figure** | **Test** | **Comparison** | **p =** |
| --- | --- | --- | --- |
| 1B | Mann Whitney test | αCD4+αCD8β vs IgG | 0.0008 |
| 1C | Kruskal-Wallis test with Dunn's multiple comparisons test | IgG vs. αCD4 | 0.0053 |
| 1C | Kruskal-Wallis test with Dunn's multiple comparisons test | IgG vs. αCD8β | 0.7697 |
| 1D | Mann Whitney test | αCD4+αCD8β vs IgG | 0.0114 |
| 1 S1E | Mann Whitney test | A2 - WT vs μMT + VP1 mAb | 0.888359 |
| 1 S2 Left | Mann Whitney test | μMT + VP1 mAb vs WT | 0.3201 |
| 1 S2 Right | Mann Whitney test | μMT + VP1 mAb vs WT | 0.281 |
| 2B center | Kruskal-Wallis test with Dunn's multiple comparisons test | IgG vs. αCD4+αCD8β | <0.0001 |
| 2B center | Kruskal-Wallis test with Dunn's multiple comparisons test | IgG vs. αCD4 | <0.0001 |
| 2B center | Kruskal-Wallis test with Dunn's multiple comparisons test | IgG vs. αCD8β | >0.9999 |
| 2B right | Mantel-Cox test with Bonferroni’s correction for multiple comparisons | IgG vs. αCD4+αCD8β | <0.001 |
| 2B right | Mantel-Cox test with Bonferroni’s correction for multiple comparisons | IgG vs. αCD4 | <0.001 |
| 2B right | Mantel-Cox test with Bonferroni’s correction for multiple comparisons | IgG vs. αCD8β | <0.001 |
| 2C | Kruskal-Wallis test with Dunn's multiple comparisons test | IgG vs. αCD4+αCD8β | <0.0001 |
| 2C | Kruskal-Wallis test with Dunn's multiple comparisons test | IgG vs. αCD4 | 0.0001 |
| 2C | Kruskal-Wallis test with Dunn's multiple comparisons test | IgG vs. αCD8β | >0.9999 |
| 2D | Kruskal-Wallis test with Dunn's multiple comparisons test | IgG vs. αCD4+αCD8β | <0.0001 |
| 2D | Kruskal-Wallis test with Dunn's multiple comparisons test | IgG vs. αCD4 | 0.0189 |
| 2D | Kruskal-Wallis test with Dunn's multiple comparisons test | IgG vs. αCD8β | >0.9999 |
| 2E | Kruskal-Wallis test with Dunn's multiple comparisons test | IgG vs. αCD4+αCD8β | <0.0001 |
| 2E | Kruskal-Wallis test with Dunn's multiple comparisons test | IgG vs. αCD4 | 0.021 |
| 2E | Kruskal-Wallis test with Dunn's multiple comparisons test | IgG vs. αCD8β | >0.9999 |
| 3B | Mann Whitney test | A2 - IgG vs VP1 mAb | 0.017185 |
| 3B | Mann Whitney test | A2.Δ292/Y294H - IgG vs VP1 mAb | 0.972766 |
| 3B | Mann Whitney test | A2.Y294D - IgG vs VP1 mAb | 0.972766 |
| 3B | Mann Whitney test | A2.Δ294 - IgG vs VP1 mAb | 0.059054 |
| 3B | Mann Whitney test | A2.Δ295 - IgG vs VP1 mAb | 0.443542 |
| 3B | Mann Whitney test | A2.D295A/Δ297 - IgG vs VP1 mAb | 0.628477 |
| 3B | Mann Whitney test | A2.D295N/Δ297 - IgG vs VP1 mAb | 0.929573 |
| 3B | Mann Whitney test | A2.V296F - IgG vs VP1 mAb | 0.972766 |
| 3C | Kruskal-Wallis test with Dunn's multiple comparisons test | A2 vs. A2.Y294D | 0.0349 |
| 3C | Kruskal-Wallis test with Dunn's multiple comparisons test | A2 vs. A2.Δ292/Y294H | 0.1035 |
| 3C | Kruskal-Wallis test with Dunn's multiple comparisons test | A2 vs. A2.Δ295 | >0.9999 |
| 3C | Kruskal-Wallis test with Dunn's multiple comparisons test | A2 vs. A2.V296F | 0.0042 |
| 3C | Kruskal-Wallis test with Dunn's multiple comparisons test | A2 vs. A2.D295A/Δ297 | >0.9999 |
| 3C | Kruskal-Wallis test with Dunn's multiple comparisons test | A2 vs. A2.D295N/Δ297 | >0.9999 |
| 3D | Kruskal-Wallis test with Dunn's multiple comparisons test | A2 vs. A2.Y294D | 0.0005 |
| 3D | Kruskal-Wallis test with Dunn's multiple comparisons test | A2 vs. A2.Δ292/Y294H | 0.0331 |
| 3D | Kruskal-Wallis test with Dunn's multiple comparisons test | A2 vs. A2.Δ295 | >0.9999 |
| 3D | Kruskal-Wallis test with Dunn's multiple comparisons test | A2 vs. A2.V296F | <0.0001 |
| 3D | Kruskal-Wallis test with Dunn's multiple comparisons test | A2 vs. A2.D295A/Δ297 | 0.0162 |
| 3D | Kruskal-Wallis test with Dunn's multiple comparisons test | A2 vs. A2.D295N/Δ297 | 0.1265 |
| 4B Center | Mann Whitney test | αCD4+αCD8β vs IgG | <0.0001 |
| 4B Right | Mantel-Cox test | αCD4+αCD8β vs IgG | 0.0001 |
| 4C | Mann Whitney test | αCD4+αCD8β vs IgG | <0.0001 |
| 4D | Mann Whitney test | αCD4+αCD8β vs IgG | <0.0001 |
| 4E | Mann Whitney test | αCD4+αCD8β vs IgG | <0.0001 |
| 5A | Mann Whitney test | A2 - IgG vs VP1 mAb | 0.00863 |
| 5A | Mann Whitney test | A2.D295N - IgG vs VP1 mAb | 0.00863 |
| 5A | Mann Whitney test | A2.Δ297 - IgG vs VP1 mAb | 0.734619 |
| 5A | Mann Whitney test | A2.D295N/Δ297 - IgG vs VP1 mAb | 0.734619 |
| 5B | One-way ANOVA test with Dunn's multiple comparisons test | A2 vs. A2.Δ297 | <0.0001 |
| 5B | One-way ANOVA test with Dunn's multiple comparisons test | A2 vs. A2.D295N | <0.0001 |
| 5B | One-way ANOVA test with Dunn's multiple comparisons test | A2 vs. A2.D295N/Δ297 | <0.0001 |
| 5D | One-way ANOVA test with Dunn's multiple comparisons test | A2 vs. A2.D295N | <0.0001 |
| 5D | One-way ANOVA test with Dunn's multiple comparisons test | A2 vs. A2.D295N/Δ297 | <0.0001 |
| 5E | One-way ANOVA test with Dunn's multiple comparisons test | A2 vs. A2.Δ297 | 0.2442 |
| 5E | One-way ANOVA test with Dunn's multiple comparisons test | A2 vs. A2.D295N | <0.0001 |
| 5E | One-way ANOVA test with Dunn's multiple comparisons test | A2 vs. A2.D295N/Δ297 | <0.0001 |
| 5F | Two-way ANOVA test with Dunn's multiple comparisons test | 24 hpi - A2 vs. A2.Δ297 | 0.9976 |
| 5F | Two-way ANOVA test with Dunn's multiple comparisons test | 24 hpi - A2 vs. A2.D295N | 0.9988 |
| 5F | Two-way ANOVA test with Dunn's multiple comparisons test | 24 hpi - A2 vs. A2.D295N/Δ297 | 0.7828 |
| 5F | Two-way ANOVA test with Dunn's multiple comparisons test | 48 hpi - A2 vs. A2.Δ297 | 0.9702 |
| 5F | Two-way ANOVA test with Dunn's multiple comparisons test | 48 hpi - A2 vs. A2.D295N | 0.9747 |
| 5F | Two-way ANOVA test with Dunn's multiple comparisons test | 48 hpi - A2 vs. A2.D295N/Δ297 | 0.2306 |
| 5F | Two-way ANOVA test with Dunn's multiple comparisons test | 72 hpi - A2 vs. A2.Δ297 | 0.9122 |
| 5F | Two-way ANOVA test with Dunn's multiple comparisons test | 72 hpi - A2 vs. A2.D295N | 0.9383 |
| 5F | Two-way ANOVA test with Dunn's multiple comparisons test | 72 hpi - A2 vs. A2.D295N/Δ297 | <0.0001 |
| 5F | Two-way ANOVA test with Dunn's multiple comparisons test | 96 hpi - A2 vs. A2.Δ297 | <0.0001 |
| 5F | Two-way ANOVA test with Dunn's multiple comparisons test | 96 hpi - A2 vs. A2.D295N | <0.0001 |
| 5F | Two-way ANOVA test with Dunn's multiple comparisons test | 96 hpi - A2 vs. A2.D295N/Δ297 | <0.0001 |
| 5H | Kruskal-Wallis test with Dunn's multiple comparisons test | A2 vs. A2.Δ297 | 0.0054 |
| 5H | Kruskal-Wallis test with Dunn's multiple comparisons test | A2 vs. A2.D295N | 0.0001 |
| 5H | Kruskal-Wallis test with Dunn's multiple comparisons test | A2 vs. A2.D295N/Δ297 | >0.9999 |
| 5 S1A | Kruskal-Wallis test with Dunn's multiple comparisons test | A2 vs. A2.D295N | 0.9739 |
| 5 S1A | Kruskal-Wallis test with Dunn's multiple comparisons test | A2 vs. A2.D295N/Δ297 | 0.0002 |
| 5 S1A | Kruskal-Wallis test with Dunn's multiple comparisons test | A2.D295N vs. A2.D295N/Δ297 | 0.0079 |
| 5 S1B | One-way ANOVA test with Dunn's multiple comparisons test | A2 vs. A2.Δ297 | 0.7742 |
| 5 S1B | One-way ANOVA test with Dunn's multiple comparisons test | A2 vs. A2.D295N | 0.6941 |
| 5 S1B | One-way ANOVA test with Dunn's multiple comparisons test | A2 vs. A2.D295N/Δ297 | <0.0001 |
| 5 S1D | One-way ANOVA test with Dunn's multiple comparisons test | A2 vs. A2.Δ297 | 0.0575 |
| 5 S1D | One-way ANOVA test with Dunn's multiple comparisons test | A2 vs. A2.D295N | <0.0001 |
| 5 S1D | One-way ANOVA test with Dunn's multiple comparisons test | A2 vs. A2.D295N/Δ297 | 0.3512 |
| 5 S1E | Mann Whitney test | A2 : - Neuraminidase vs + Neuraminidase | 0.00863 |
| 5 S1E | Mann Whitney test | A2.Δ297: - Neuraminidase vs + Neuraminidase | 0.00863 |
| 5 S1E | Mann Whitney test | A2.D295N: - Neuraminidase vs + Neuraminidase | 0.00863 |
| 5 S1E | Mann Whitney test | A2.D295N/Δ297: - Neuraminidase vs + Neuraminidase | 0.00863 |
| 5 S1F | Mann Whitney test | A2 : - Neuraminidase vs + Neuraminidase | 0.00863 |
| 5 S1F | Mann Whitney test | A2.Δ297: - Neuraminidase vs + Neuraminidase | 0.00863 |
| 5 S1F | Mann Whitney test | A2.D295N: - Neuraminidase vs + Neuraminidase | 0.00863 |
| 5 S1F | Mann Whitney test | A2.D295N/Δ297: - Neuraminidase vs + Neuraminidase | 0.00863 |
| 6A | Mann Whitney test | A2 vs. A2.D295N/Δ297 | 0.1296 |
| 6D Left | Mann Whitney test | A2 vs. A2.D295N/Δ297 | <0.0001 |
| 6D Right | Mann Whitney test | A2 vs. A2.D295N/Δ297 | <0.0001 |
| 6E | Mann Whitney test | A2 vs. A2.D295N/Δ297 | <0.0001 |
| 6H | One-way ANOVA test with Dunn's multiple comparisons test | Sham vs. A2 | 0.0003 |
| 6H | One-way ANOVA test with Dunn's multiple comparisons test | Sham vs. A2.D295N/Δ297 | <0.0001 |
| 6H | One-way ANOVA test with Dunn's multiple comparisons test | A2 vs. A2.D295N/Δ297 | <0.0001 |
